# Drug use prevention: factors associated with program implementation in Brazilian urban schools

**DOI:** 10.1186/s12889-018-5242-y

**Published:** 2018-03-07

**Authors:** Ana Paula Dias Pereira, Zila M. Sanchez

**Affiliations:** 1 Programa de Pós-graduação em Saúde Coletiva, Departamento de Medicina Preventiva, Universidade Federal de São Paulo, São Paulo, Brazil; 2 Department of Preventive Medicine, Epidemiology, Universidade Federal de São Paulo, Rua Botucatu, 740, 4th floor, São Paulo, SP 04023-900 Brazil

## Abstract

**Background:**

A school is a learning environment that contributes to the construction of personal values, beliefs, habits and lifestyles, provide convenient settings for the implementation of drug use prevention programs targeting adolescents, who are the population group at highest risk of initiating drug use. The objective of the present study was to investigate the prevalence of factors associated with implementing drug use prevention programs in Brazilian public and private middle and high urban schools.

**Methods:**

The present population-based cross-sectional survey was conducted with a probability sample of 1151 school administrators stratified by the 5 Brazilian administrative divisions, in 2014. A close-ended, self-reported online questionnaire was used. Logistic regression analysis was used to identify factors associated with implementing drug use prevention programs in schools.

**Results:**

A total of 51.1% of the schools had adopted drug use prevention programs. The factors associated with program implementation were as follows: belonging to the public school network; having a library; development of activities targeting sexuality; development of “Health at School Program” activities; offering extracurricular activities; and having an administrator that participated in training courses on drugs.

**Conclusions:**

The adoption of drug use prevention practices in Brazilian schools may be expanded with greater orchestration of schools through specialized training of administrators and teachers, expansion of the School Health Program and concomitant development of the schools’ structural and curricular attributes.

## Background

School-based prevention programs for adolescent drug use have been developed and implemented in several countries [1]. A school is a learning environment that contributes to the construction of personal values, beliefs, habits and lifestyles at a time when adolescents are more susceptible to reflect on such issues, and this can directly affect the social production of health [2]. From this perspective, schools provide convenient settings for the implementation of drug use prevention programs targeting adolescents, who are the population group at highest risk of initiating drug use [3].

Preventive interventions implemented at the beginning of and throughout adolescence have the potential to reduce the rates of drug use and associated problems in adulthood [4, 5]. This has been demonstrated by efficacy and effectiveness studies of school-based prevention programs in the last decades [6–8]. Economic evaluation analyses of the implementation of such programs indicate that school-based preventive interventions produce a savings of US$ 38 for every dollar invested [9].

Studies investigating the presence of preventive programs in schools and their characteristics as well as those seeking to identify possible facilitators and barriers to program implementation are not common but contribute to decision-making that facilitates the future introduction of preventive programs in the school curriculum [5]. In some developed countries, studies on the prevalence of factors associated with the implementation of drug use prevention programs in schools are much more developed than those in Latin American countries such as Brazil. The current discussions in developed countries extend the paradigm by focusing on the level of scientific evidence of school-based programs. For example, some studies were conducted in the United States to establish the prevalence of evidence-based drug prevention programs in schools [10–13] and to investigate the factors associated with the adoption of programs based on scientific evidence of efficacy or effectiveness [14]. The results showed that less than half of American schools (47%) had an evidence-based drug use prevention program, while in the past decade, most of the schools chose to adopt programs with no established efficacy or effectiveness [12]. Regarding the factors associated with the adoption of a curricular program with scientific evidence of efficacy or effectiveness in American schools, it was found that the larger time dedicated by the leaders in the activities for drug abuse prevention in their schools [15] and the availability of financial resources seem to be associated with the decision to implement programs in schools [16]. Another factor associated with the implementation of programs in schools is the assistance that schools receive from government agencies and the supply of informational materials on prevention [14].

Brazil is a country with continental dimensions and is currently one of the most unequal societies, as shown by a Gini index (calculation used to measure social inequality) of 0.5. Approximately 97.4% of the population aged 6 to 14 years old and 87.7% of the population aged 15 to 19 years old attend school, representing almost universal schooling coverage of children and younger adolescents [17]. In a population-based epidemiological survey conducted with more than 50,000 students aged 10 to 19 years of age from 27 Brazilian state capitals, 60.5% of the sample reported having consumed alcohol at some point in their lives, while 25.5% reported having used illegal drugs and 16.9% reported having used tobacco, thus pointing to a severe social and public health problem within the school environment [18]. While drug use by Brazilian students in the past 30 years has been well documented [18], little is known about the implementation of drug use prevention programs in the country. There is no governmental or scientific information about the existence of these programs in Brazilian schools or even worse, to date this topic has not been studied in developing countries.

Public policies have been formulated in Brazil to target the health of students, including the “Health at School Program” (Programa Saúde na Escola – PSE). The aim of the PSE is to contribute to the integral education of public school students through promoting healthy eating, a culture of peace and humans rights, prevention and reduction of alcohol, drug, and tobacco use and sexual health. However, there are no reports of the implementation of such actions and their characteristics in Brazilian schools [17, 19].

Considering the need to assess the magnitude of the implementation of school-based drug use prevention programs in Brazil, the present study investigated the prevalence of factors associated with the implementation of such programs in public and private middle (grades 6 to 9) and high (grades 10 to 12) schools.

This study examined the demographic characteristics of schools, school structure and curriculum activities. That starting point was the hypothesis that these factors influence the decision making for implementing prevention of drug use in Brazilian schools programs.

## Methods

The present population-based cross-sectional survey was conducted with a probability sample of private and public school administrators from the South, Southeast, North, Northeast and Central-West regions, which are the 5 Brazilian administrative divisions according to the Brazilian Institute of Geography and Statistics (Instituto Brasileiro de Geografia e Estatística – IBGE) [20]. For the purposes of the present study, principals, pedagogical supervisors and prevention program coordinators were considered school administrators.

### Participants

The study sample was composed of private and public school administrators chosen through random selection, using the lottery method, of schools stratified per administrative division. That means that the sample was self-weighted, considering that participants and replacements were kept proportional according to the sampling universe of each region and school network. Only middle and high schools in urban areas were considered. Schools from rural areas were not included due to the possible lack of internet connection. The National Register of Basic Education Schools, 2012 School Census, was provided by the National Institute for Educational Studies and Research (Instituto Nacional de Estudos e Pesquisas Educacionais - INEP). Thus, the sample universe included 52,065 schools.

The sample size was calculated based on the finite sample universe (*n* = 52,065), a confidence level of 95%, an absolute error of 3% and a response distribution of 50% (as there were no previous data on the prevalence of prevention programs in Brazilian schools); thus, the required sample was 1046 schools.

Considering potential losses that are common in studies in which data are collected via the Internet [21, 22], the number of participating schools was increased to 2090 to ensure that the minimum estimated sample size would be met despite losses and replacements.

The sample of school administrators was chosen because studies have shown that school leaders, principals and pedagogical coordinators are the main decision-makers regarding the adoption of a drug prevention program [16, 23].

### Instrument and variables

A self-report closed-ended questionnaire was answered anonymously over the Internet. The questionnaire included 45 questions that evaluated respondent’s characteristics; school characteristics; health education at school; respondent’s training in drug issues; and processes of decision-making relative to the adoption of drug use prevention programs. Some questions were taken from the questionnaire used in American schools as described by [24], while others were specifically developed to investigate the characteristics of the Brazilian programs; the comprehensibility of these questions was assessed in the pilot study.

The variables related to school structure were extracted from the National Register of Basic Education Schools, 2012 School Census database, which are official governmental data provided by the INEP.

#### Outcome variable

The outcome variable was “having a drug use prevention program incorporated into the everyday school routine and in the annual school program” (yes/no).

#### Explanatory variables

The explanatory variables were divided across the following 5 domains: respondent’s demographic data, school demographic data, school structure, curriculum activities performed at the school and organizational factors related to decision-making regarding the adoption of drug use prevention programs.

The respondent demographic variables considered were as follows: gender (male or female); age (categorized in the following ranges: 20–29, 30–39, 40–49, 50–59 and 60–69 years of age); educational level (secondary school, incomplete higher education, complete higher education, graduate education – master/doctoral degree); position (principal, pedagogical supervisor, prevention program coordinator, other); and length of time at the present position, at the school and in education (in years).

The school characteristics were evaluated based on the following variables: school network (public or private), region (Southeast, South, Northeast, North or Center-West), location (capital or interior of the state) and school size (small ≤800 students, medium = 801 to 1600 students, large > 1600 students).

The school structure was analyzed based on whether it had the following (yes/no): computer laboratory, science laboratory, reading room and library.

The curriculum activities were evaluated based on the following binary variables (yes/no): the school develops activities targeting topics related to health, sexuality, eating habits, PSE activities and extracurricular activities, and the school tests new curricula, programs, innovative teaching practices and respondents participate in courses on drugs.

### Procedures

The data were collected during the 2014 school year after a pilot study was performed in 2013 with 263 private and public school administrators from São Paulo [25].

All administrators were invited to participate in the study via e-mails sent to the school’s e-mail address using SurveyMonkey software, which sent all messages at once. If no response was received after 4 e-mails, the potential participants were called by telephone and invited to participate in the study, and any questions they had were answered. A total of 1555 telephone calls were made.

### Data analysis

In the descriptive analysis, the qualitative variables were summarized as absolute frequencies, percentages and 95% confidence intervals (CI). The data corresponding to numerical variables are expressed as the median and interquartile range. The chi-square test was used in the initial comparison of categorical variables. Logistic regression models were fit to identify the factors associated with the implementation of drug use prevention programs, with the outcome variable being having a prevention program. Explanatory variables related to the respondents’ demographic data, school demographic data, school structure and curriculum activities were analyzed.

The variables were first analyzed separately using univariate logistic regression models. Next, a logistic regression model was fitted. The first model included all variables with *p* < 0.20 on univariate analysis. Variables without statistical significance were excluded in each domain until the final model, which included only statistically significant variables. The significance level for the hypothesis tests and the final model was set to 5%. The goodness of fit of the final regression model was assessed by use of the Hosmer-Lemeshow test. The results are expressed as the odds ratio (OR) with the corresponding 95% CI. All analyses were performed in Stata 13.

## Results

**Fig. 1:**
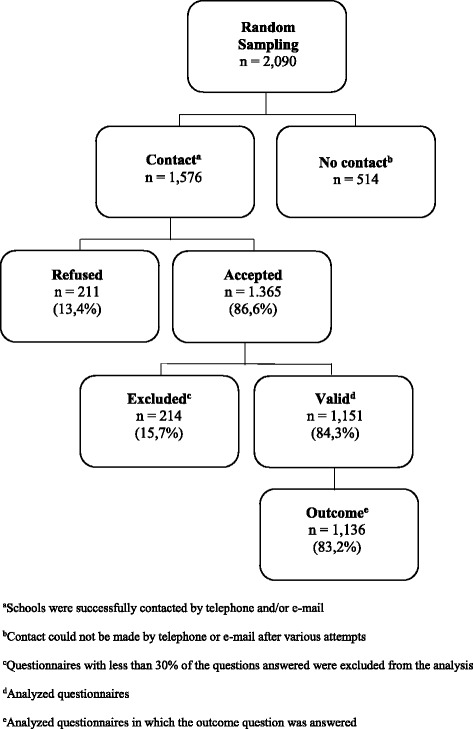
Flowchart representing the sample of Brazilian schools that participated in the study

A total of 2090 schools were invited to participate, but 514 did not respond to the invitation. Among the 1576 schools that responded to the invitation, 211 (13,4%) refused to participate. Of the 1151 valid questionnaires, 1136 administrators responded to the outcome question and were included in the analysis (Fig. 1). Table 1 describes respondent characteristics. Most of the participants were school principals, female, and 40 to 49 years of age and had a high educational level. The length of work at the current school varied from less than one to 42 years (median = 8 years). More than half of the respondents had at least 20 years of experience in education and had worked in their current position for more than 4 years.

**Table 1:** Respondents’ (school administrators) demographic characteristics and length of professional activity, Brazil, 2014 (*n* = 1136)

	Has a drug use prevention program									
	Yes			No			Total			
	*n = 580*			*n = 556*			*n = 1.136*			
Variables	*n*	%	95% CI	*n*	%	95% CI	*n*	%	95% CI	*p-value*
Gender^a^										
Female	440	76,0	72,3–79,7	405	73,0	69,1–76,6	845	74,5	71,9–77,0	0,243
Male	139	24,0	20,6–27,7	150	27,0	23,4–30,9	289	25,5	23,0–28,1	
Age										
20–29 years	18	3,1	1,8–4,9	34	6,1	4,3–8,4	52	4,6	3,4–6,0	0,060
30–39 years	164	28,3	24,6–32,1	175	31,5	27,6–35,5	339	29,8	27,2–32,6	
40–49 years	249	42,9	38,9–47,1	216	38,8	34,8–43,0	465	40,9	38,1–43,9	
50–59 years	124	21,4	18,1–24,9	114	20,5	17,2–24,1	238	21,0	18,6–23,4	
60–69 years	25	4,3	2,8–6,3	17	3,1	1,8–4,8	42	3,7	2,7–5,0	
Educational level^a^										
Secondary school	2	0,3	0,04–0,1	7	1,3	0,5–2,6	9	0,8	0,4–1,5	0,042
Incomplete higher education	9	1,6	0,7–2,9	11	2,0	1,0–3,5	20	1,8	1,1–2,7	
Complete higher education	115	19,9	16,7–23,3	140	25,2	21,6–29,0	255	22,5	20,1–25,0	
Non-degree graduate education (specialization)	402	69,4	65,5–73,2	363	65,3	61,2–69,2	765	67,4	64,6–70,1	
Graduate education (master or doctorate)	51	8,8	6,6–11,4	35	6,3	4,4–8,6	86	7,6	6,1–9,3	
Position^b^										
Principal	281	54,8	50,3–59,1	219	47,4	42,8–52,1	500	51,3	48,1–54,45	0,012
Pedagogical supervisor	182	35,5	31,3–39,8	210	45,5	40,8–50,1	392	40,2	27,1–43,4	
Prevention program coordinator	37	7,2	5,1–9,8	22	4,8	3,1–7,1	59	6,1	4,6–7,7	
Other	13	2,5	1,4–4,3	11	2,4	1,2–4,2	24	2,5	1,6–3,6	
Length of time (years)	Mean	DP	95% CI	Mean	DP	95% CI				
Current position	6,8	6,1	6,3–7,3	5,9	5,8	5,4–6,3				0,008
Current school	10,4	8,0	9,8–11,1	9,0	7,5	8,4–9,6				0,002
Education	20,2	8,4	19,5–20,8	18,2	8,8	17,4–18,9				0,001

^a^Missing data did not exceed 1%

^b^13% of this variables responses were missing

A total of 51.1% (95% CI: 48.1–54.0) of schools had incorporated drug use prevention activities into the everyday school routine and pedagogical plan. The demographic and structural characteristics and curriculum activities of the schools, stratified by the presence or absence of drug use prevention programs, are presented in Table 2. Schools from all 5 Brazilian regions participated in the study, though public schools, small schools and schools located in the interior of the states predominated. Most schools had computer laboratories (63.6%), while less than half had a library, science laboratory or reading room. The curriculum of activities performed at the schools included activities regarding student health. The data indicated a high prevalence of activities targeting health, sexuality and eating habits.

**Table 2:** School demographic characteristics, structure and curriculum activities, Brazil, 2014 (*n* = 1136)

	Has a drug use prevention program									
	Yes			No			Total			
	*n = 580*			*n = 556*			*n = 1.136*			
Variables	*n*	%	95% CI	*n*	%	95% CI	*n*	%	95% CI	*p-value*
Demographic characteristics										
School network										
Private	115	19,8	16,7–23,3	140	25,2	21,6–29,0	255	22,4	20,1–25,0	0,031
Public	465	80,2	76,7–83,3	416	74,8	71,0–78,4	881	77,6	75,0–79,9	
Region										
Southeast	299	51,6	47,4–55,7	220	39,6	35,5–43,8	519	45,7	42,8–48,6	< 0,001
South	95	16,4	13,5–19,7	66	11,9	9,9–14,8	161	14,2	12,2–16,3	
Northeast	94	16,2	13,3–19,5	190	34,2	30,2–38,3	284	25,0	22,5–27,6	
North	37	6,4	4,5–8,7	40	7,2	5,2–9,7	77	6,8	5,4–8,4	
Central-West	55	9,5	7,2–12,2	40	7,2	5,2–9,7	95	8,4	6,8–10,1	
Size^a^										
Small	403	69,5	65,6–73,2	394	70,9	66,9–74,6	797	70,2	67,3–73,0	0,818
Medium	147	25,3	21,8–29,1	132	23,7	20,3–27,5	279	24,6	22,1–27,2	
Large	30	5,2	3,5–7,3	30	5,4	3,7–7,6	60	5,3	4,0–6,7	
Location										
Capital	118	20,5	17,3–24,1	110	20,0	16,7–23,6	228	20,3	17,9–22,7	0,828
Interior	457	79,5	75,9–82,7	440	80,0	76,4–83,3	897	79,7	77,1–82,2	
School structure										
Computer laboratory (Yes)	405	70,6	66,6–74,2	310	56,4	52,1–60,5	715	63,6	60,7–66,4	< 0,001
Science laboratory (Yes)	151	26,3	22,7–30,1	109	19,8	16,6–23,4	260	23,1	20,7–25,7	< 0,001
Reading room (Yes)	153	26,7	23,1–30,5	139	25,3	21,7–29,1	292	26,0	23,4–28,6	0,597
Library (Yes)	304	53,0	48,8–57,1	199	36,2	32,2–40,2	503	44,8	41,8–47,7	< 0,001
Curriculum activities										
Health (Yes)	573	98,8	97,5–99,5	524	94,2	92,0–96,0	1097	96,6	95,3–97,5	< 0,001
Sex education (Yes)	553	95,3	93,3–96,9	480	86,3	83,2–89,1	1033	90,9	89,1–92,5	< 0,001
Eating habits (Yes)	545	94,0	91,7–95,8	469	84,4	81,1–87,3	1014	89,3	87,3–91,0	< 0,001
PSE^b^ (Yes)	244	42,1	38,0–46,2	160	28,8	25,0–32,7	404	35,6	32,8–38,4	< 0,001
Extracurricular activities^c^ (Yes)	455	78,9	75,3–82,1	373	67,1	63,0–71,0	828	73,1	70,4–75,6	< 0,001
Novel teaching practices (Yes)	507	89,1	86,2–91,5	341	79,3	75,2–83,0	848	84,9	82,5–87,0	< 0,001
Attended training course^d^ (Yes)	430	74,1	70,4–77,7	309	55,6	51,3–59,8	739	65,1	62,2–67,8	< 0,001

^a^Small (up to 800 students); medium (801 to 1600 students); large (more than 1600 students)

^b^Health at School Program, Federal Government

^c^Activities performed outside school hours

^d^Respondents participate in courses on drugs

Table 3 describes the factors associated with the implementation of drug use prevention programs. The data suggest that public schools, compared with private schools, had 38% greater odds of having a drug use prevention program (OR = 1.38; 95% CI 1.00–1.91). The Northeastern region exhibited the lowest odds of having a prevention program compared to the Southeastern region, which is the region with the largest population (OR = 0.35; 95% CI 0.24; 0.49). The schools’ physical structure was also associated with the presence of prevention programs. Schools with libraries had twice the odds of having a prevention program as those without libraries (OR = 1.73; 95% CI 1.28; 2.35). Conducting activities targeting sexuality (OR = 2,34; 95% CI 1.43; 3.81), offering extracurricular activities (OR = 2.00; 95% CI 1.48; 2.64), performing PSE-oriented activities (OR = 1.98; 95% CI 1.48; 2.69) and having administrators who had attended training courses on drugs (OR = 1.97; 95% CI 1.50; 2.58) were positively associated with the presence of drug use prevention programs.

**Table 3:** Organizational factors related to decision-making surrounding the adoption of drug use prevention programs, Brazil, 2014 (*n* = 1136)

	Univariate regression			Multivariate regression		
Variables	OR	95% CI	*p*-value	OR	95% CI	*p*-value
School demographic characteristics						
School network						
Private	1,00	–	–	1,00	–	–
Public	1,36	1,03–1,80	0,031	1,38	1,00–1,91	0,047
Region						
Southeast	1,00	–	–	1,00	–	–
South	1,06	0,74–1,52	0,754	0,59	0,39–0,90	0,015
Northeast	0,36	0,27–0,49	< 0,001	0,35	0,24–0,49	< 0,001
North	0,68	0,42–1,10	0,116	0,56	0,33–0,96	0,350
Central-West	1,01	1,14–1,61	0,959	0,72	0,45–1,16	0,183
School structure						
Computer laboratory (Yes)	1,85	1,45–2,37	< 0,001	–	–	–
Science laboratory (Yes)	1,44	1,09–1,91	0,010	–	–	–
Library (Yes)	1,98	1,56–2,52	< 0,001	1,73	1,28–2,35	< 0,001
Curriculum activities						
Health (Yes)	5,00	2,19–11,4	< 0,001	–	–	–
Sex education (Yes)	3,24	2,06–5,12	< 0,001	2,34	1,43–3,81	0,001
Eating habits (Yes)	2,99	1,91–4,36	< 0,001	–	–	–
PSE (Yes)	1,80	1,40–2,30	< 0,001	1,98	1,49–2,64	< 0,001
Extracurricular activities (Yes)	1,83	1,40–2,39	< 0,001	2,00	1,48–2,69	< 0,001
Novel teaching practices (Yes)	2,13	1,50–3,03	< 0,001	–	–	–
Attended training course – Administrators (Yes)	2,29	1,78–2,94	< 0,001	1,97	1,50–2,58	< 0,001

The Hosmer–Lemeshow goodness-of-fit test *p* value for the adoption of a drug use prevention program in the school at the final logistic model (Table 3) was 0.420, indicating that the model was adequately adjusted.

## Discussion

The present study showed that half of the schools participating in the study utilize a drug use prevention program as part of the school curriculum. The factors associated with implementing drug use prevention programs in schools included the type of school network; Brazilian administrative division; presence of a library in the school; performance of activities targeting sexuality; availability of extracurricular activities (“outside school hours”); performance of PSE-oriented activities; and having administrators who had attended training courses on drugs.

Few international studies have assessed the prevalence of drug use prevention programs that are included as a part of the middle and high school curriculum. The United States, where three-fourths of schools include these programs in the curriculum, has a greater prevalence of such programs than Brazil. This prevalence is the result of political effort, investment and scientific dissemination, which in addition to stimulating the adoption of prevention programs in schools, also contribute to improving the quality of program implementation [12, 26]. Thus, identifying the factors associated with the implementation of drug use prevention programs is fundamental to producing knowledge that increases the prevalence of program implementation. With accurate understanding of the factors that favor the adoption of drug use prevention programs in schools, government and school administrators can formulate guidelines and provide resources to increase the availability of such programs in schools; this implementation is likely to reduce the use of alcohol, tobacco and other drugs among school-attending adolescents [27, 28]. However, studies on the prevalence of factors associated with the implementation of prevention programs do not report program quality and effectiveness; other types of studies are needed for this purpose. This proviso is important because the implementation of a prevention program does not necessarily mean that drug use will effectively be reduced. However, it indicates that the school community is investing effort toward that goal.

The greater participation of public schools in the present study is in accordance with the distribution of public schools in Brazil, as currently 73% of schools are public [29].

In addition, our data showed that public schools, compared with private schools, implemented drug use prevention programs more often. Nevertheless, there is evidence that wealthier adolescents are at high risk for alcohol and drug use in Brazil [30]. The latest Brazilian national survey, which was conducted among 50,890 students at public and private schools from all 27 state capitals, found a greater proportion of students in private schools who used drugs [18]. Data on the prevalence of drug use among students should be effectively disseminated to the administrators of private schools to help dispel the myth that the prevalence of drug use among students from the higher social classes is low, and it may also draw attention to this problem in private schools [14]. It is possible that administrators in wealthier schools may not perceive the need for implementing drug abuse prevention activities [31].

Another factor associated with the demographic characteristic of the school is the region of Brazil that the school is located. Schools in the Northeastern region were less likely to have a prevention program compared to the Southeast region, which is the region with the highest population. Thus, the present study evidences inequalities, among the regions of the country, in relation to the preventive actions to the use of drugs in the Brazilian schools and indicates the need of more significant investment in governmental actions that reach the schools of the country as a whole. Similar findings were identified in another study evaluating school settings in Brazil [32].

Schools’ physical structure also seems to be associated with the implementation of prevention programs. Schools with libraries were more likely to adopt drug use prevention programs than schools without libraries. This finding can suggests that the administrators of such schools are concerned with integral education and thus promote the development of reading habits by students and facilitate their access to information. Information was shown to be protective against drug use [33], and consequently, depending on their collections, school libraries may contribute to the education of students regarding drug use.

Some aspects of the school curriculum were also associated with the adoption of prevention programs. For example, adherence to the federal government’s PSE almost doubled the odds of implementing a drug use prevention program. Within the context of the PSE, schools, aided by healthcare professionals, develop health promotion activities including the encouragement of healthy eating and physical activity and the prevention of drug use [19]. Some evidence indicates that implementing the PSE effectively favors the development of activities for drug use prevention. However, the PSE only operates in public schools, and thus, there is a need to extend its coverage to private schools. In contrast, 28.8% of the schools under PSE orientation have not yet adopted drug use prevention programs, which goes against PSE guidelines and points to the need to supervise what schools offer to their students.

Development of activities for sex education was associated with adoption of drug use prevention programs, suggesting that these schools target more than one high-risk behavior and likely integrate information on drug use with information on sexual behavior. This is relevant because some scientific evidence indicates that high-risk sexual behavior among adolescents is associated with binge drinking (defined as drinking more than five alcoholic drinks on one occasion) and the use of illegal drugs [34]. In addition, because drug use is associated with other high-risk behaviors, the efficacy of programs that integrate these topics is usually greater than that of programs that focus on drug use alone [34, 35].

Regarding the activities available at the schools, one of the main findings of the present study is that drug use prevention programs were more frequently adopted in schools that offered extracurricular activities compared to those that did not. According to a study conducted with 2903 Polish adolescents aged 13 and 14 years old, participation in sports and religious and artistic activities was protective against alcohol consumption [36]. Researchers suggest that this protection derives from the fact that extracurricular activities fill students’ free time and allow them to learn social roles and develop skills that contribute to their positive development [36–38], and they may complement the possible protective effects of school-based prevention programs.

Attending training courses was associated with the implementation of prevention programs in schools. In Brazil, the National Secretariat on Drug Policies (Secretaria Nacional de Políticas sobre Drogas - SENAD), at the Ministry of Justice, provides free training courses for education professionals on drug use prevention in schools [39]. In our current study, we found that schools whose administrators participated in drug training courses tended to implement prevention programs more frequently. The training received in such courses by administrators may have alerted to the need to adopt prevention programs or it could be that people who felt strongly about drug education and prevention took a course in that topic and helped implement their school’s inclusion of prevention activities. Alternatively, they may have taken a position at a school that already had implemented prevention activities and then took a course in this topic to help them understand it.

The present study had some limitations. First, data collection through the Internet resulted in a low participation rate among the school principals (approximately 65.3%), and non-participating schools differed from those of participating schools on Brazilian Region and type of school. There was larger losses in North and Central-West region of the country and among private schools. Additionally, there might have been some degree of information bias due to the use of a self-reported questionnaire that could be subjectively interpreted by each respondent. Furthermore, as the study was cross-sectional, we could not infer causality between the associated factors and program implementation because the temporal relationship between them could not be identified.

Finally, the present study was not designed to evaluate the quality of the implemented prevention programs but rather to quantify their presence in a probability sample of Brazilian schools. Thus, future studies should be specifically designed to assess the efficacy and effectiveness of these programs. The results may serve to orient public policies favoring the implementation of preventive programs in developing countries such as Brazil.

Based on the data presented here, we recommend some actions that may contribute to the implementation of future programs in a system-perspective, such as: Production and dissemination of scientific knowledge that may stimulate the adoption of new programs in the country; Favorable political and legislative structure for the implementation of drug prevention programs in the school context; Law enforcement of preventive policies; Training in prevention of drug use by health and education professionals; Commitment of the leaders in relation to the practice of prevention to the use of drugs in the schools; Investment in infrastructure and resources throughout the country; Expansion of public policies for private schooling; Government actions consistent with partnerships with researchers in the area of prevention, to assist in decision making for implementation of a drug prevention program.

## Conclusion

The results of the present study suggest that adoption of drug use prevention measures by schools can increase given greater orchestration of the schools through the specialized training of administrators and teachers and the promotion of consistent collaboration between the health and education sectors. This could be encouraged by increasing schools’ participation in programs such as the PSE, with concomitant development of their structural and curricular aspects.

There are several lessons that can be learned from the present study, specially to empower directors and stakeholders who plan to implement a program to prevent drug use in their school or school district. The influence of the school administration on adoption of drug prevention programs was evident in this study. Schools whose administrators/directors had participated in training courses on drugs tend to implement prevention programs more frequently on their schools, suggesting the need to expand the public distribution of such programs in Brazil and in countries with similar educational structure. Besides, some aspects of the school curriculum have been associated with the adoption of prevention programs, such as the concurrent implementation of sexual education activities, availability of extracurricular activities (“outside school hours”) and conducting activities developed by the governmental School Health Program.

## Abbreviations

CAPES: Brazilian Federal Agency for Support and Evaluation of Higher Education
CI: Confidence Interval
CNPq: National Council of Scientific and Technological Development
IBGE: Brazilian Institute of Geography and Statistics
INEP: National Institute for Educational Studies and Research
OR: Odds ratio
PSE: Health at School Program
SENAD: National Secretariat on Drug Policies
